# Impact of right ventricle-pulmonary artery coupling in patients undergoing transcatheter aortic valve implantation

**DOI:** 10.1007/s10554-024-03165-0

**Published:** 2024-06-28

**Authors:** Lígia Fernandes Mendes, Mariana Brandão, Silvia O. Diaz, Marta Catarina Almeida, António S. Barros, Francisca Saraiva, José Ribeiro, Alberto Rodrigues, Pedro Braga, Ricardo Fontes Carvalho, Francisco Sampaio

**Affiliations:** 1 Faculdade de Medicina da Universidade do Porto, Porto, Portugal; 2 Centro Hospitalar de Vila Nova de Gaia/Espinho, EPE, Porto, Portugal; 3 Cardiovascular R&D Centre - UnIC@RISE, Department of Surgery and Physiology, Faculty of Medicine, University of Porto, Porto, Portugal

**Keywords:** TAVI, Right ventricle-pulmonary artery coupling, Aortic stenosis, TAPSE/PASP ratio

## Abstract

Right ventricle-pulmonary artery (RV-PA) coupling has been linked to clinical outcomes in patients with severe aortic stenosis (AS) undergoing transcatheter valve implantation (TAVI). However, the best timing for prognostic assessment remains uncertain. Our aim was to determine the impact of RV longitudinal function parameters and RV-PA coupling on mortality in patients undergoing TAVI.  Retrospective, single center, analysis including patients with AS who underwent TAVI between 2007 and 2021. Echocardiographic evaluation was performed before, shortly after the procedure, and during follow-up. RV-PA uncoupling was defined as a TAPSE/PASP ratio<0.55 (severe RV uncoupling was defined as TAPSE/PASP ratio<0.32. The effect of RV parameters on all-cause mortality up to 12 months was assessed.  Among the 577 patients included, pre-procedural TAPSE/PASP ratio data were available for 205. RV-PA uncoupling was present in 113 patients (55.1%), with severe uncoupling observed in 31 (15.1%). Within the first 12 months after TAVI, 51 patients (9%) died. Severe RV-PA uncoupling was associated with mortality in univariable Cox regression; however, this association was lost after adjusting for EuroSCORE II. A significant association was found between the TAPSE/PASP ratio (per 0.1-unit increase) after the procedure and the primary endpoint (HR: 0.73; 95% CI: 0.56, 0.97; p=0.029). Higher postprocedural PASP (HR: 1.04; 95% CI: 1.02, 1.06; p<0.001 was also associated with all-cause mortality.  V-PA uncoupling and PASP after TAVI are associated with all-cause mortality in patients and may be valuable for patient selection and for planning post-procedural care.

**Supplementary Information:**

The online version contains supplementary material available at 10.1007/s10554-024-03165-0.

## Introduction

Aortic stenosis (AS) is an increasingly prevalent valve disease, due to the ageing population [1]. Transcatheter aortic valve implantation (TAVI) is currently considered the preferred mode of intervention among individuals after the 7th decade of life or those deemed high-risk or unsuitable for surgery [2, 3].

In recent years, many observational studies have focused on the role of right ventricular function on the outcome of patients with severe AS undergoing TAVI [4–6]. Right ventricle-pulmonary artery (RV-PA) coupling reflects the ability of RV systolic performance to deal with a given afterload and can be estimated by the ratio of tricuspid annular plane systolic excursion (TAPSE) to pulmonary artery systolic pressure (PASP) [7–10]; the lower the TAPSE/PASP ratio, the worse the RV-PA [4, 7, 8]. The presence and severity of RV-PA uncoupling has been strongly associated with worse clinical outcomes [10, 11].

However, the best timing for assessing the TAPSE/PSAP ratio for prognostic purposes remains unclear among previous studies.

This study aimed to (1) determine the impact of RV longitudinal function parameters and RV-PA coupling on mortality in patients undergoing TAVI and (2) evaluate the evolution of RV echocardiographic parameters before the procedure, immediately after the procedure and at follow-up.

## Materials and methods

### Study population

We conducted a single-center retrospective analysis of patients who underwent TAVI for the treatment of severe native AS, between August 2007 and December 2021.

Baseline characteristics, echocardiographic parameters, procedural, and outcome data were collected from clinical records. Mortality at 12 months was assessed by collecting the date of death from the clinical files.

### Echocardiographic evaluation

All patients underwent echocardiographic evaluation before TAVI, in the immediate post-procedural period (before discharge, up to 96 h after valve implantation) and during follow-up (up to 12 months after procedure).

Longitudinal function parameters of the right ventricle assessed included TAPSE and S-wave tissue Doppler velocity of the tricuspid annulus (RV S’). Standard thresholds for RV dysfunction were used: TAPSE < 17 mm and RV S’ <10 cm/s [12, 13].

TR was assessed qualitatively and graded on a scale of trace to mild to severe [12, 14]. PASP was calculated by the peak velocity of tricuspid regurgitation plus the estimated right atrial pressure, based on the inferior vena cava diameter and collapsibility index (+ 5/10/15 mmHg) [12].

TAPSE/PASP ratio was used as a surrogate of RV-PA coupling [10]; TAPSE/PASP ratio < 0.55 was used to define RV-PA uncoupling, built on previous studies [8, 11, 13]. A TAPSE/PASP ratio value < 0.32 mm/mmHg was used to define severe RV-uncoupling [10, 13, 15].

### Study endpoints

The primary endpoint of the study was the effect of pre- and post-procedural RV parameters (TAPSE, PASP, TAPSE/PASP ratio and RV S’) on all-cause mortality up to 12-months after TAVI.

Secondary endpoint was the longitudinal change in RV echocardiographic parameters from pre-procedure, immediately after and at the first follow-up visit (within 12 months after TAVI).

### Statistical analysis

Patient characteristics were summarized as counts and percentages for categorical variables and median and IQR (25th to 75th percentiles) for continuous variables. Patients were then stratified according to their TAPSE/PASP ratio measured before TAVI (used here as a surrogate for RV uncoupling). The *p trend* was computed to assess the linear association between the TAPSE/PASP ratio and each characteristic, by fitting a linear regression with the TAPSE/PASP value as the dependent variable and each characteristic as an independent variable.

The effect of RV parameters (measured prior and immediately after TAVI) on mortality up to 12-months after procedure, was explored through Cox proportional hazard model, univariately and adjusted to EuroSCORE II. The latter score was considered for model adjustment as it is a cardiac surgery risk score that sums clinical, analytical, and echocardiographic parameters. All Hazard Ratios (HR) along 95% confidence intervals, p-values and C-index were reported. Proportional hazard assumption of the Cox models was tested using Schoenfeld residuals.

The longitudinal change of RV parameters, from before TAVI, immediately after and at the 1st follow-up, was assessed through non-parametric Friedman test. This test was used as RV parameters did not follow a normal distribution in all time-points (confirmed through Shapiro-Wilk test and visual inspection of histograms and Q-Q plots). The Friedman test effect size (i.e., Kendall’s W), a standardized metric of the effect, was also computed.

A *p* < 0.05 was deemed statistically significant. All statistical analysis and plots were done with R statistical software, version 4.1.2 [16–19]. .

### Ethics

The study was conducted in accordance with the Declaration of Helsinki and was approved by the local Ethics Committee. Informed consent was waived considering the retrospective nature of the study.

## Results

### Baseline characteristics

A total of 615 patients underwent TAVI during the study period. Of these, 38 were excluded due to unavailable baseline echocardiographic data. Among the 577 patients included in this analysis, the assessment of pre- and post-procedural TAPSE/PSAP ratio was possible for 205 and 188 patients, respectively. During follow-up echocardiographic evaluation, the relationship was determined in 121 patients (Flowchart 1). The total number of missing values for all variables, at each time, is shown in Supplementary Table 1.

**Flowchart 1 ?:**
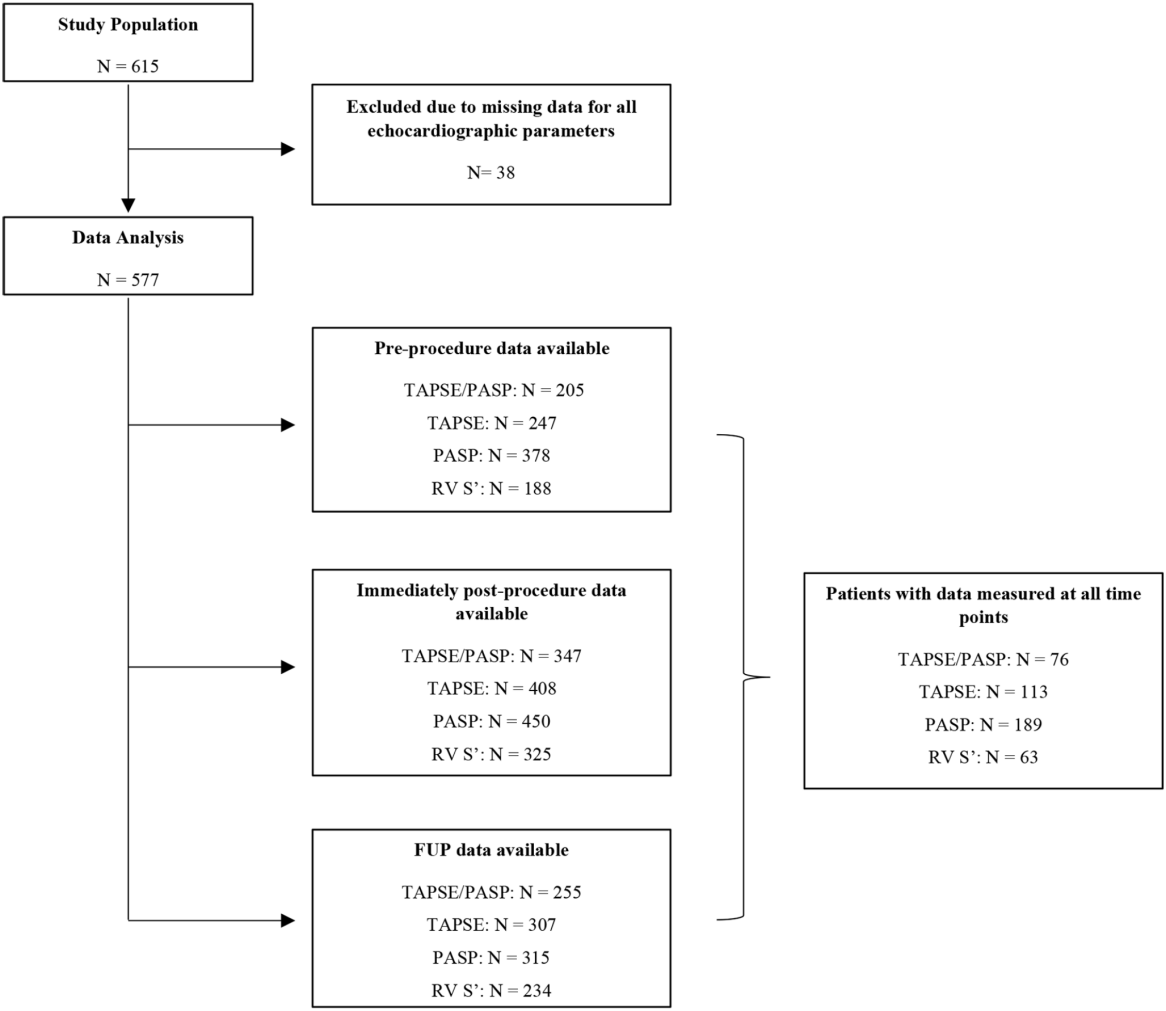
Exclusion criteria and follow-up evaluation. ETT = transthoracic echocardiogram; FUP = follow-up; PASP = pulmonary artery systolic pressure; Post = immediately post-procedure; TAPSE = tricuspid annular plane systolic excursion

Patient’s demographics and baseline characteristics are summarized in Table 1. 54% of the patients were female. Patients included in the study population had a median age of 81 years (IQR: 76–85) and a median EuroSCORE II of 3.5.

Approximately half of the patients (48%) were in New York Heart Association (NYHA) class III/IV at the time of intervention, with a median left ventricular ejection fraction of 55% (IQR: 47–60). Moderate to severe TR was found in 10.6% of the sample.

**Table 1:** Baseline characteristics of the patient population

Characteristics	*N* = 577	
Sex		
Women	312 (54%)	
Age	81 [76, 85]	
BMI, kg/m^2^	26.7 [23.9, 29.4]	
NYHA functional class III or IV	248 (48%)	
Current or former smoker	81 (17%)	
Hypertension	418 (82%)	
Diabetes	211 (39%)	
Dyslipidemia	189 (73%)	
COPD	83 (15%)	
AF/flutter	132 (32%)	
Prior MI	179 (33%)	
Previous stroke	56 (10%)	
LV ejection fraction, %	55 [47, 60]	
Mean aortic gradient, mmHg	46 [39, 55]	
AVA, cm^2^	0.70 [0.60, 0.80]	
EuroScore II score, %	3.5 [2.1, 5.7]	
TR		
None		79 (14%)
Minimal		279 (48%)
Mild		158 (27%)
Moderate to severe		61 (10.6%)
PASP, mmHg	39 [32, 50]	
RV TAPSE, mm	20.0 [18.0, 22.0]	
TAPSE/PASP	0.50 [0.37, 0.66]	
RV S´, cm/s	11.15 [9.80, 13.00]	
Post procedure TR		
None	102 (19%)	
Minimal	256 (48%)	
Mild	122 (23%)	
Moderate to severe	54 (10%)	
Post procedure PASP, mmHg	35 [29, 42]	
Post procedure RV TAPSE, mm	20.0 [17.0, 22.0]	
Post procedure TAPSE/PASP	0.57 [0.42, 0.71]	
Post procedure RV S´, cm/s	11.50 [10.00, 13.80]	
FUP TR		
None	81 (21%)	
Minimal	161 (41%)	
Mild	106 (27%)	
Moderate to severe	43 (11%)	
FUP PASP, mmHg	35 [30, 43]	
FUP RV TAPSE, mm	20.0 [18.0, 23.0]	
FUP TAPSE/PASP	0.57 [0.46, 0.69]	
FUP RV S´, cm/s	11.25 [10.00, 13.00]	

Continuous variables are shown as median (IQR) and categorical as n (%)

AF = atrial fibrillation; AVA = aortic valve area; BMI = body mass index; COPD = chronic obstructive pulmonary disease; FUP = follow-up; LV = left ventricle; MI = myocardial infarction; NYHA = New York Heart Association; PASP = pulmonary artery systolic pressure; Post = immediately post-procedure; TAPSE = tricuspid annular plane systolic excursion; TR = tricuspid regurgitation

### RV parameters

The median baseline TAPSE/PASP ratio of the entire population was 0.50 (IQR: 0.37–0.66). Immediately after the procedure, the TAPSE/PASP ratio increased to a median value of 0.57 (IQR: 0.42–0.71) and remained stable during follow-up. (Table 1).

Patients’ characteristics from baseline to 1st follow-up according to the baseline TAPSE/PASP ratio, are shown in Table 2. RV-PA uncoupling, defined by TAPSE/PASP ratio < 0.55 was present in 113 patients (55.1%); among these, 31 (15.1%) showed severe uncoupling (TAPSE/PSAP < 0.32).

Patients with lower TAPSE/PSAP ratio had a higher prevalence of NYHA functional class ≥ III (*p* = 0.005) and atrial fibrillation (AF) or flutter (*p* < 0.001). The lower the ratio TAPSE/PASP, the lower LV ejection fraction (LVEF) (*p* < 0.001) (Table 2).

Patients with lower baseline TAPSE/PASP ratios showed worse longitudinal function indexes at baseline (TAPSE 21 mm vs. 19 mm vs. 17 mm, *p* < 0.001; RV S’ 11.6 cm/s vs. 11.0 cm/s vs. 9.0 cm/s, *p* < 0.001); on the contrary, PASP was higher (32 mmHg vs. 47 mmHg vs. 66 mmHg, *p* < 0.001). At 1-year follow-up, patients with lower baseline TAPSE/PASP ratios had higher PASP **(**Table 2).

**Table 2:** Baseline characteristics of the study group according to TAPSE/PASP ratio

Characteristic	TAPSE/PASP > 0,55 *N* = 92	TAPSE/PASP= [0,32:0,55] *N* = 82	TAPSE/PASP < 0,32 *N* = 31	*p*-trend
Sex				0.4
Female	48 (52%)	50 (61%)	13 (42%)	
Age	82 [75, 85]	82 [77, 86]	77 [71, 82]	0.7
BMI, kg/m^2^	26.0 [23.7, 29.0]	27.6 [24.0, 30.9]	25.9 [23.3, 29.1]	0.4
NYHA functional class III or IV	33 (44%)	36 (46%)	23 (79%)	0.005
Current or former smoker	16 (22%)	12 (17%)	4 (15%)	0.077
Hypertension	63 (82%)	66 (86%)	21 (75%)	0.5
Diabetes	27 (34%)	32 (40%)	14 (48%)	0.3
Dyslipidemia	18 (75%)	17 (89%)	9 (90%)	0.3
COPD	10 (13%)	11 (14%)	4 (14%)	0.8
AF/flutter	13 (23%)	17 (45%)	10 (71%)	< 0.001
Prior MI	26 (33%)	16 (20%)	8 (28%)	0.11
Previous stroke	7 (8.9%)	9 (11%)	3 (11%)	0.4
LV ejection fraction, %	57 [53, 62]	55 [44, 61]	43 [27, 55]	< 0.001
Mean aortic gradient, mmHg	46 [41, 55]	48 [39, 55]	45 [39, 57]	0.6
AVA, cm^2^	0.70 [0.60, 0.80]	0.70 [0.60, 0.80]	0.70 [0.60, 0.70]	0.2
EuroScore II score, %	3.0 [1.9, 4.7]	3.6 [2.3, 5.0]	6.5 [3.7, 15.8]	< 0.001
Pre-procedure RV characteristics				
TR				< 0.001
Minimal	74 (80%)	31 (38%)	4 (13%)	
Mild	18 (20%)	37 (45%)	12 (39%)	
Moderate to severe	0 (0%)	14 (17%)	15 (48%)	
PASP, mmHg	32 [27, 35]	47 [41, 53]	66 [56, 80]	< 0.001
RV TAPSE, cm	21.0 [20.0, 24.0]	19.0 [17.2, 21.8]	17.0 [14.0, 19.0]	< 0.001
RV S´	11.60 [10.17, 13.00]	11.00 [9.90, 12.55]	9.00 [7.85, 10.90]	< 0.001
Post-procedure RV characteristics	TAPSE/PASP > 0,55 *N* = 83	TAPSE/PASP= [0,32:0,55] *N* = 77	TAPSE/PASP < 0,32 *N* = 28	p-trend
TR				< 0.001
None	14 (17%)	8 (11%)	1 (3.5%)	
Minimal	51 (61%)	34 (44%)	7 (25%)	
Mild	14 (17%)	24 (31%)	12 (43%)	
Moderate to severe	4 (5%)	11 (14%)	8 (28.5%)	
PASP, mmHg	30 [26, 34]	38 [31, 46]	48 [39, 55]	< 0.001
RV TAPSE, cm	20.0 [18.0, 22.5]	18.5 [17.0, 21.2]	17.0 [14.0, 19.0]	< 0.001
TAPSE/PASP	0.68 [0.59, 0.83]	0.50 [0.37, 0.66]	0.36 [0.30, 0.43]	< 0.001
RV S´	12.45 [10.53, 13.97]	10.50 [9.60, 11.75]	10.40 [8.80, 12.20]	< 0.001
FUP RV characteristics	TAPSE/PASP > 0,55 *N* = 59	TAPSE/PASP= [0,32:0,55] *N* = 38	TAPSE/PASP < 0,32 *N* = 24	p-trend
TR				< 0.001
None	10 (17%)	4 (11%)	3 (12%)	
Minimal	31 (52%)	16 (42%)	4 (17%)	
Mild	14 (24%)	13 (34%)	7 (29%)	
Moderate to severe	4 (7%)	5 (13%)	10 (42%)	
PASP, mmHg	32 [26, 39]	36 [32, 45]	54 [37, 65]	< 0.001
RV TAPSE, cm	21.0 [18.0, 24.0]	20.0 [18.0, 22.0]	19.5 [17.3, 21.8]	0.4
TAPSE/PASP	0.62 [0.54, 0.70]	0.60 [0.42, 0.69]	0.47 [0.29, 0.55]	< 0.001
RV S´	12.10 [9.68, 13.93]	12.40 [10.20, 14.00]	10.40 [9.80, 12.00]	0.076

Continuous variables are shown as median (IQR) and categorical as n (%)

AF= atrial fibrillation; AVA = aortic valve area; BMI = body mass index; COPD = chronic obstructive pulmonary disease; FUP = follow-up; LV = left ventricle; MI = myocardial infarction; NYHA = New York Heart Association; PASP = pulmonary artery systolic pressure; Post = immediately post-procedure; TAPSE = tricuspid annular plane systolic excursion; TR = tricuspid regurgitation

### Clinical outcomes

Fifty-one (9%) patients died within the first 12 months after TAVI.

A higher pre-procedural PASP [Hazard Ratio (HR): 1.02; 95% CI: 1.00, 1.04; *p* = 0.028)] and the presence of severe RV-PA uncoupling (HR: 3.21; 95% CI: 1.19, 8.67; *p* = 0.022) were associated with all-cause mortality in the univariable Cox regression. However, this association was not observed after adjusting to EuroSCORE II (Table 3).

There was a significant association of TAPSE/PASP ratio after procedure (per 0.1-unit increase) and the primary endpoint (HR: 0.73; 95% CI: 0.56, 0.97; *p* = 0.029). A higher post-procedural PASP (HR: 1.04; 95% CI: 1.02, 1.06; *p* < 0.001) was associated with an all-cause mortality in the univariable Cox regression; this was maintained after adjusting to EuroSCORE II (Table 3).

There was no statistically significant association between mortality up to 12 months after TAVI and the RV function assessed by RV S’ or TAPSE, either pre- or post-procedural (Table 3).

**Table 3:** Cox proportional hazard models for all-cause mortality

				Unadjusted							Adjusted to EuroSCORE II (%)					
	Characteristic	*N*			Event *N*	HR	95% CI	*p*-value	C-index	*N*		Event *N*	HR	95% CI	*p*-value	C-index
Pre-procedure	PASP, mmHg	378			35	1.02	1.00, 1.04	**0.028**	0.603	344		30	1.02	1.00, 1.05	0.062	0.614
	RV TAPSE, cm	247			20	0.97	0.86, 1.09	0.6	0.525	223		16	0.96	0.83, 1.10	0.5	0.616
	TAPSE/PASP (per 0.1 increase)	205			17	0.80	0.61, 1.05	0.10	0.604	184		14	0.81	0.58, 1.12	0.2	0.622
	RV S´	188			15	0.94	0.76, 1.16	0.6	0.569	172		13	0.95	0.74, 1.22	0.7	0.683
	TAPSE < 17 mm	247			20	2.16	0.72, 6.45	0.2	0.549	223		16	1.75	0.49, 6.29	0.4	0.649
	TAPSE/PASP < 0.32	205			17	3.21	1.19, 8.67	**0.022**	0.603	184		14	2.59	0.72, 9.35	0.15	0.652
	TAPSE/PASP < 0.55	205			17	1.16	0.44, 3.06	0.8	0.518	184		14	1.09	0.35, 3.39	0.9	0.632
Post-procedure	PASP, mmHg	450			33	1.04	1.02, 1.06	**< 0.001**	0.639	410		30	1.04	1.02, 1.06	**< 0.001**	0.664
	RV TAPSE, cm	408			26	0.95	0.86, 1.05	0.3	0.574	366		21	0.95	0.84, 1.06	0.4	0.640
	TAPSE/PASP (per 0.1 increase)	347			21	0.77	0.61, 0.97	**0.030**	0.643	314		18	0.73	0.56, 0.97	**0.029**	0.667
	RV S´	325			20	0.95	0.80, 1.13	0.6	0.549	289		16	0.96	0.79, 1.16	0.7	0.612
	TAPSE < 17 mm	408			26	1.51	0.57, 4.00	0.4	0.528	366		21	1.25	0.40, 3.98	0.7	0.609
	TAPSE/PASP < 0.32	347			21	2.99	1.09, 8.15	**0.033**	0.570	314		18	3.16	1.07, 9.39	**0.038**	0.602
	TAPSE/PASP < 0.55	347			21	2.97	1.15, 7.65	**0.024**	0.629	314		18	3.80	1.23, 11.8	**0.021**	0.675

CI= confident interval; HR = hazard ratio; PASP = pulmonary artery systolic pressure; Post = immediately post-procedure; RV = right ventricle; RV S? = S-wave tissue Doppler velocity of the tricuspid annulus; TAPSE = tricuspid annular plane systolic excursion

### Longitudinal analysis of RV parameters

**Fig. 1:**
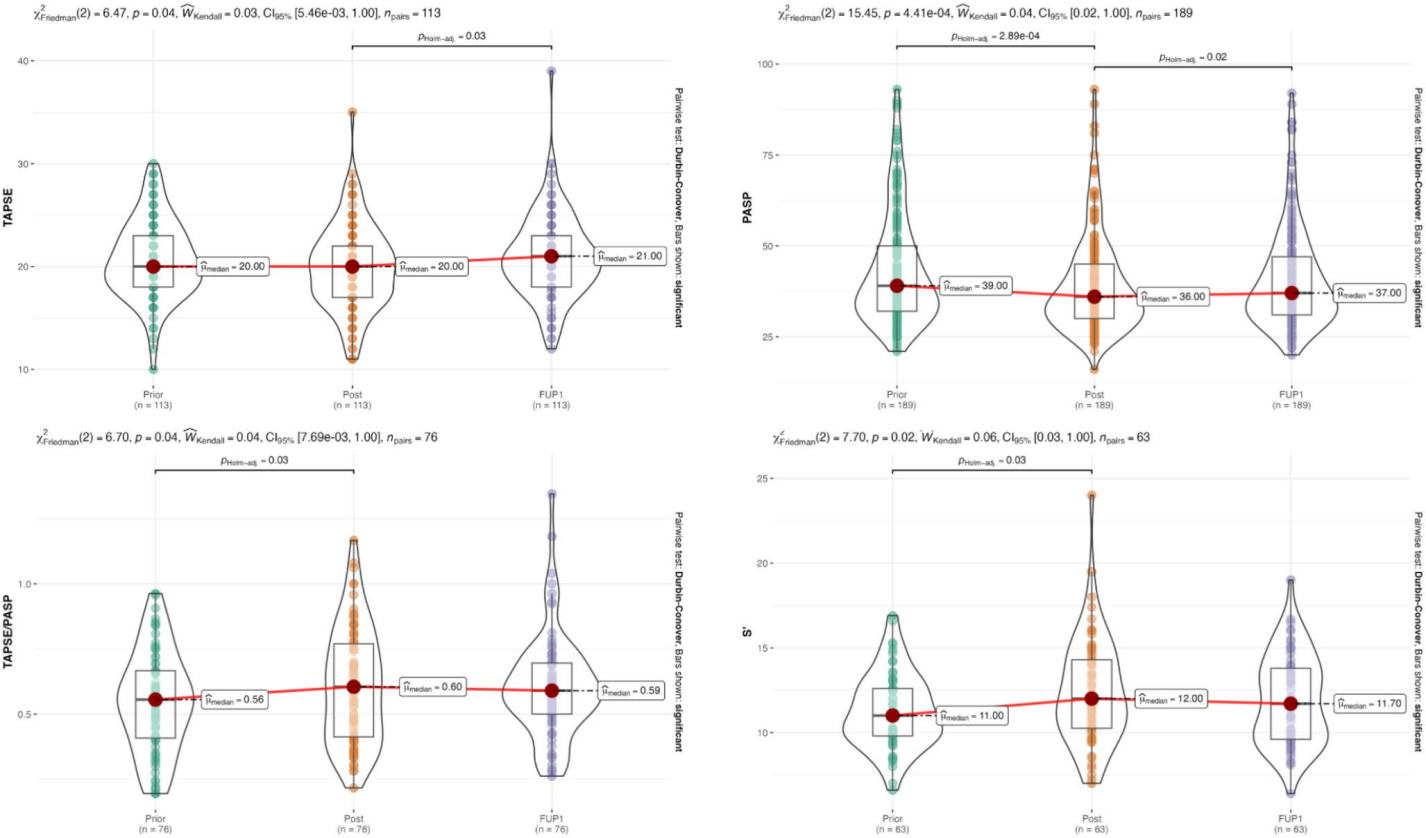
Variation of RV parameters from baseline to 1 year after the procedure. Violin plots of all measurements and their median value. In these plots, each point represents one patient, only patients with the 3 measurements available were included. FUP ? follow-up up to 1 year after procedure; PASP = pulmonary artery systolic pressure; Post = immediately post-procedure; RV = right ventricle; RV S? = S-wave tissue Doppler velocity of the tricuspid annulus; TAPSE = tricuspid annular plane systolic excursion

The longitudinal variation of RV parameters is shown in Fig. 1.

PASP values in all evaluation were available for 189 participants. There was a statistically significant decrease in PASP (p-value < 0.01) from pre- (median = 39 mmHg) to post-TAVI (median = 36 mmHg) to first FUP (median = 37 mmHg).

TAPSE values were available for 113 participants at all time points. There was a statistically significant increase in RV TAPSE (p-value = 0.04) from pre- (median = 20 mm) to post-TAVI (median = 20 mm) to first FUP (median = 21 mm).

Sixty-three individuals had S’ measurements across the 3 echo evaluations A statistically significant increase in RV S’ (p-value = 0.02) from pre- (median = 11.00 cm/s) to post-TAVI (median = 12.00 cm/) to first FUP (median = 11.70 cm/s) was observed.

TAPSE/PASP ratio values at all time points were available for 76 participants. There was a statistically significant increase in TAPSE/PASP ratio (p-value = 0.04) from pre- (median = 0.56) to post-TAVI (median = 0.60) to first FUP (median = 0.59).

## Discussion

We found that: (1) RV-PA uncoupling, particularly after the procedure, was independently associated with mortality at 12 months in patients undergoing TAVI; (2) there was an improvement in TAPSE/PASP ratio, TAPSE and PASP after TAVI and (3) higher post-procedural PASP was associated with mortality.

The prognostic impact of RV-PA uncoupling in patients undergoing TAVI has been previously reported [4, 8, 11, 20]. Sultan et al. found that, in a single center retrospective analysis of 457 patients undergoing TAVI between 2011 and 2016, TAPSE/PASP either as a continuous variable or as quartiles was associated with higher mortality, even after adjusting for several variables [20]. Similarly, Adamo et al., reported that a TAPSE/PASP ratio < 0.36 mm/mmHg was associated with an increased risk of mortality after TAVI, independently of the surgical risk score [4]. In a sub analysis of the PARTNER 3 trial, baseline RV-PA uncoupling was also associated with worse clinical outcomes at 2 years, but the best cut-off of the TAPSE/PSAP ratio for predicting the primary outcome across the cohort was found to be 0.55 mm/mmHg [8, 21]. We found that both a higher pre-procedural PASP and the presence of severe RV-PA uncoupling (defined as a ratio < 0.32) were associated with all-cause mortality in the univariate analysis; however, this association was lost after adjusting to EuroSCORE II. Differences in patients’ characteristics (the PARTNER 3 trial included only low-risk patients) or follow-up length may account for these conflicting results. The observation that patients with worse pre-procedural RV-PA uncoupling were more symptomatic (NYHA class ≥ III), had higher prevalence of AF/flutter, lower LVEF, more severe TR and worse RV function - some of which are considered in the EUROSCORE – is in line with other studies [4, 11, 20] and may explain this finding. Also, 55% of our patients had RV-PA uncoupling, of which approximately 15% had severe uncoupling. One of the key findings of our study, which is corroborated by others, is that the improvement of RV-PA coupling is mainly due to a reduction in pulmonary pressure, reflecting post-capillary pulmonary hypertension as the main pathophysiological process [8, 9, 11]. On the other hand, Meucci et al., reported that, in 900 patients undergoing TAVI in 2 centers, post-procedural RV-PA uncoupling was independently associated with mortality, whereas pre-procedural uncoupling was not [11]. Our results are in line with these, since we also found a significant association between the post-TAVI TAPSE/PASP ratio, as a continuous variable, and all-cause mortality, even after adjusting for EUROSCORE II. Hence, the improvement in RV/PA coupling after TAVI, rather than RV/PA uncoupling which was very prevalent before the procedure and probably explains the lack of statistical significance for this variable, seem to be the main prognostic marker in these patients.

We found that estimated PASP decreased, and RV longitudinal function indices and RV-PA coupling improved after TAVI and that these changes were detectable shortly (up to 96 h) after the procedure. These findings were also reported by others [4, 8], although the impact of TAVI on RV longitudinal function indices and the main driver of the improvement of RV-PA coupling is not clear [8, 9]. Differences in the timing of the echocardiographic assessment may partially explain conflicting results. Notwithstanding, the persistence of higher post-procedural PASP seems to be an important prognostic finding, being independently associated with mortality in our study, even in multivariate analysis. This was also reported by other authors [22–24].

This is a single-center retrospective analysis, including a limited number of patients and events, which may have influenced the statistical power to detect differences. Furthermore, there are losses to follow-up and missing data in several variables, particularly due to the absence of TAPSE measurements or the reduction in TR (precluding estimation of PASP) after the procedure; this is, however, common to most studies on this subject. We did not provide an optimal cutoff for the TAPSE/PASP ratio which would have been clinically relevant and could add to the existing literature. However, since dividing continuous variables into distinct categories based on arbitrary cut-offs could result in loss of information, reduced statistical power, and potentially biased outcomes, we decided not to perform this analysis.

The estimation of PASP from the peak tricuspid regurgitation velocity may be underestimated in patients with severe tricuspid regurgitation; however, the number of patients with this condition in our study was small. Additional RV parameters, such as FAC3D RVEF and speckle tracking data were not available. Additional information on the cause of death (cardiovascular vs. non-cardiovascular), as well as other outcomes such as hospitalizations were not available.

TAVI decreases PASP and improves RV-PA coupling in patients with severe AS. Our study suggests that both RV-PA uncoupling and PASP after TAVI are predictors of all-cause mortality. These findings may be helpful to improve risk stratification and useful to plan post-procedural care in patients with severe native AS submitted to TAVI.

## Electronic supplementary material

Below is the link to the electronic supplementary material.

Supplementary Material 1

## Data Availability

No datasets were generated or analysed during the current study.

## References

[1] Ancona R, Pinto SC (2020) Epidemiology of aortic valve stenosis (AS) and of aortic valve incompetence (AI): is the prevalence of AS/AI similar in different parts of the world? E-J Cardiol Pract, *18*(10). https://www.escardio.org/Journals/E-Journal-of-Cardiology-Practice/Volume-18/epidemiology-of-aortic-valve-stenosis-as-and-of-aortic-valve-incompetence-ai

[2] Vahanian A, Beyersdorf F, Praz F et al (2022) 2021 ESC/EACTS guidelines for the management of valvular heart disease: developed by the Task Force for the management of valvular heart disease of the European Society of Cardiology (ESC) and the European Association for Cardio-thoracic surgery (EACTS). Eur Heart J 43(7):561–632. 10.1093/EURHEARTJ/EHAB395 34453165 10.1093/EURHEARTJ/EHAB395

[3] Members WC, Otto CM, Nishimura RA et al (2021) 2020 ACC/AHA Guideline for the management of patients with Valvular Heart Disease: executive summary: a report of the American College of Cardiology/American Heart Association Joint Committee on Clinical Practice guidelines. J Am Coll Cardiol 77(4):450–500. 10.1016/j.jacc.2020.11.035 33342587 10.1016/j.jacc.2020.11.035

[4] Adamo M, MacCagni G, Fiorina C et al Prognostic value of right ventricle to pulmonary artery coupling in transcatheter aortic valve implantation recipients. J Cardiovasc Med (Hagerstown Md), *23*(9), 615–622. 10.2459/JCM.0000000000001336 10.2459/JCM.0000000000001336 35994710

[5] Asami M, Stortecky S, Praz et al (2019) Prognostic value of right ventricular dysfunction on clinical outcomes after transcatheter aortic valve replacement. JACC Cardiovasc Imaging 12(4):577–587. 10.1016/J.JCMG.2017.12.015 29454762 10.1016/J.JCMG.2017.12.015

[6] Testa L, Latib A, De Marco et al (2016) The failing right heart: implications and evolution in high-risk patients undergoing transcatheter aortic valve implantation. EuroIntervention: J EuroPCR Collab Working Group Interventional Cardiol Eur Soc Cardiol 12(12):1542–1549. 10.4244/EIJ-D-15-00148 10.4244/EIJ-D-15-00148 27998847

[7] Brener MI, Lurz P, Hausleiter J et al (2022) Right ventricular-pulmonary arterial coupling and Afterload Reserve in patients undergoing transcatheter tricuspid valve repair. J Am Coll Cardiol 79(5):448–461. 10.1016/J.JACC.2021.11.031/35115101 10.1016/J.JACC.2021.11.031/

[8] Cahill TJ, Pibarot P, Yu X et al (2022) Impact of right ventricle-pulmonary artery coupling on clinical outcomes in the PARTNER 3 trial. JACC Cardiovasc Intervent 15(18):1823–1833. 10.1016/J.JCIN.2022.07.005 10.1016/J.JCIN.2022.07.005 36137685

[9] Lillo R, Graziani F, Ingrasciotta G et al (2022) Right ventricle systolic function and right ventricle-pulmonary artery coupling in patients with severe aortic stenosis and the early impact of TAVI. Int J Cardiovasc Imaging 38(8):1761–1770. 10.1007/S10554-022-02569-0 35230568 10.1007/S10554-022-02569-0

[10] Tello K, Wan J, Dalmer A et al (2019) Validation of the tricuspid annular plane systolic Excursion/Systolic pulmonary artery pressure ratio for the Assessment of Right Ventricular-Arterial Coupling in severe pulmonary hypertension. Circ Cardiovasc Imaging 12(9). 10.1161/CIRCIMAGING.119.009047 10.1161/CIRCIMAGING.119.009047 PMC7099862 31500448

[11] Meucci MC, Malara S, Butcher SC et al (2023) Evolution and prognostic impact of right ventricular-pulmonary artery coupling after transcatheter aortic valve replacement. JACC Cardiovasc Intervent 16(13):1612–1621. 10.1016/J.JCIN.2023.05.003 10.1016/J.JCIN.2023.05.003 37438027

[12] Galderisi M, Cosyns B, Edvardsen T et al (2017) Standardization of adult transthoracic echocardiography reporting in agreement with recent chamber quantification, diastolic function, and heart valve disease recommendations: an expert consensus document of the European Association of Cardiovascular Imaging. Eur Heart J Cardiovasc Imaging 18(12):1301–1310. 10.1093/EHJCI/JEX244 29045589 10.1093/EHJCI/JEX244

[13] Humbert M, Kovacs G, Hoeper MM et al (2022) 2022 ESC/ERS guidelines for the diagnosis and treatment of pulmonary hypertension. Eur Respir J 61(1):46. 10.1183/13993003.00879-2022 10.1183/13993003.00879-2022 36028254

[14] Lancellotti P, Tribouilloy C, Hagendorff A et al (2013) Recommendations for the echocardiographic assessment of native valvular regurgitation: an executive summary from the European Association of Cardiovascular Imaging. Eur Heart J - Cardiovasc Imaging 14(7):611–644. 10.1093/EHJCI/JET105 23733442 10.1093/EHJCI/JET105

[15] Tello K, Axmann J, Ghofrani HA et al (2018) Relevance of the TAPSE/PASP ratio in pulmonary arterial hypertension. Int J Cardiol 266:229–235. 10.1016/J.IJCARD.2018.01.053 29887454 10.1016/J.IJCARD.2018.01.053

[16] Patil I (2021) Visualizations with statistical details: the ggstatsplot approach. J Open Source Softw 6(61):3167. 10.21105/JOSS.03167 10.21105/JOSS.03167

[17] Sjoberg DD, Whiting K, Curry M et al (2021) Reproducible Summary tables with the Gtsummary Package. R J 13(1):570–580. 10.32614/RJ-2021-053 10.32614/RJ-2021-053

[18] T, T. (n.d.). *A Package for Survival Analysis in R. R package version 3.5-5*. (2023) https://cran.r-project.org/package=survival

[19] Team RC (2021) *R: A language and environment for statistical computing. R Foundation for Statistical Computing, Vienna, Austria*

[20] Sultan I, Cardounel A, Abdelkarim I et al (2018) Right ventricle to pulmonary artery coupling in patients undergoing transcatheter aortic valve implantation. Heart 105(2):117–121. 10.1136/HEARTJNL-2018-313385 30093545 10.1136/HEARTJNL-2018-313385 PMC6320315

[21] Pibarot P, Salaun E, Dahou A et al (2020) Echocardiographic results of Transcatheter Versus Surgical aortic valve replacement in low-risk patients. Circulation 141(19):1527–1537. 10.1161/CIRCULATIONAHA.119.044574 32272848 10.1161/CIRCULATIONAHA.119.044574

[22] Alushi B, Beckhoff F, Leistner D et al (2019) Pulmonary hypertension in patients with severe aortic stenosis: prognostic impact after transcatheter aortic valve replacement: pulmonary hypertension in patients undergoing TAVR. JACC Cardiovasc Imaging 12(4):591–601. 10.1016/J.JCMG.2018.02.015 29680341 10.1016/J.JCMG.2018.02.015

[23] Miyamoto J, Ohno Y, Kamioka N et al (2022) Impact of Periprocedural Pulmonary Hypertension on outcomes after Transcatheter aortic valve replacement. J Am Coll Cardiol 80(17):1601–1613. 10.1016/J.JACC.2022.08.757 36265955 10.1016/J.JACC.2022.08.757

[24] Ujihira K, Kohmoto T, Gimelli G et al (2020) The impact of increased pulmonary arterial pressure on outcomes after transcatheter aortic valve replacement. Catheterization Cardiovasc Interventions: Official J Soc Cardiac Angiography Interventions 96(7):E723–E734. 10.1002/CCD.28862 10.1002/CCD.28862 32243048

