# Supplementary material for: Impact of right ventricle-pulmonary artery coupling in patients undergoing transcatheter aortic valve implantation
Source: Int J Cardiovasc Imaging. 2024 Jun 28;40(8):1745–53. doi: 10.1007/s10554-024-03165-0 (PMC11401781; doi:10.1007/s10554-024-03165-0)
Supplement: Supplementary file 1 — Supplementary Material 1 [file 10554_2024_3165_MOESM1_ESM.docx]

**Supplementary Table 1: Absolute and relative frequencies of missing values per variable.**

AF = atrial fibrillation; AVA = aortic valve area; BMI = body mass index; COPD = chronic obstructive pulmonary disease; FUP = follow-up; LV = left ventricle; MI = myocardial infarction; NYHA = New York Heart Association; PASP = pulmonary artery systolic pressure; Post = immediately post-procedure; TAPSE = tricuspid annular plane systolic excursion; TR = tricuspid regurgitation.

|  | N | % |
| --- | --- | --- |
| Sex | 0 | 0.0% |
| Age | 0 | 0.0% |
| BMI, kg/m^2^ | 29 | 5.0% |
| NYHA functional class III/IV | 65 | 11.3% |
| Current former smoker | 108 | 18.7% |
| Hypertension | 67 | 11.6% |
| Diabetes | 34 | 5.9% |
| Dyslipidemia | 317 | 54.9% |
| COPD | 37 | 6.4% |
| AF/flutter | 169 | 29.3% |
| Prior MI | 36 | 6.2% |
| Previous stroke | 38 | 6.6% |
| LV ejection fraction, % | 218 | 37.8% |
| Mean aortic gradient, mmHg | 68 | 11.8% |
| AVA, cm^2^ | 90 | 15.6% |
| EuroScore II score, % | 58 | 10.1% |
|  | | |
| Pre-procedure RV characteristics |  |  |
| TR | 0 | 0.0% |
| PASP, mmHg | 199 | 34.5% |
| RV TAPSE, cm | 330 | 57.2% |
| TAPSE/PASP | 372 | 64.5% |
| RV S´ | 389 | 67.4% |
|  | | |
| Post-procedure RV characteristics |  |  |
| TR | 43 | 7.5% |
| PASP, mmHg | 127 | 22.0% |
| RV TAPSE, cm | 169 | 29.3% |
| TAPSE/PASP | 230 | 39.9% |
| RV S´ | 252 | 43.7% |
|  | | |
| FUP RV characteristics |  |  |
| TR | 186 | 32.2% |
| PASP, mmHg | 262 | 45.4% |
| RV TAPSE, cm | 270 | 46.8% |
| TAPSE/PASP | 322 | 55.8% |
| RV S´ | 343 | 59.4% |
